# *TODRA*, a lncRNA at the *RAD51* Locus, Is Oppositely Regulated to *RAD51*, and Enhances RAD51-Dependent DSB (Double Strand Break) Repair

**DOI:** 10.1371/journal.pone.0134120

**Published:** 2015-07-31

**Authors:** Inbal Gazy, David A. Zeevi, Paul Renbaum, Sharon Zeligson, Lital Eini, Dana Bashari, Yoav Smith, Amnon Lahad, Michal Goldberg, Doron Ginsberg, Ephrat Levy-Lahad

**Affiliations:** 1 Human Genetics, Hebrew University Medical School, Jerusalem, Israel; 2 Medical Genetics Institute, Shaare Zedek Medical Center, Jerusalem, Israel; 3 Department of Genetics, Alexander Silberman Institute of Life Sciences, Hebrew University of Jerusalem, Jerusalem, Israel; 4 The Mina and Everard Goodman Faculty of Life Science, Bar Ilan University, Ramat Gan, Israel; 5 Genomic Data Analysis Unit, Hebrew University Medical School, Jerusalem, Israel; 6 Department of Family Medicine, Hebrew University Medical School, Jerusalem, Israel; 7 Clalit Health Services, Jerusalem, Israel; University Medical Center Hamburg-Eppendorf, GERMANY

## Abstract

Expression of *RAD51*, a crucial player in homologous recombination (HR) and DNA double-strand break (DSB) repair, is dysregulated in human tumors, and can contribute to genomic instability and tumor progression. To further understand *RAD51* regulation we functionally characterized a long non-coding (lnc) RNA, dubbed *TODRA* (Transcribed in the Opposite Direction of *RA* *D51*), transcribed 69bp upstream to *RAD51*, in the opposite direction. We demonstrate that *TODRA* is an expressed transcript and that the *RAD51* promoter region is bidirectional, supporting *TODRA* expression (7-fold higher than RAD51 in this assay, p = 0.003). *TODRA* overexpression in HeLa cells induced expression of *TPIP*, a member of the TPTE family which includes PTEN. Similar to PTEN, we found that TPIP co-activates E2F1 induction of *RAD51*. Analysis of E2F1's effect on the bidirectional promoter showed that E2F1 binding to the same site that promotes *RAD51* expression, results in downregulation of *TODRA*. Moreover, *TODRA* overexpression induces HR in a RAD51-dependent DSB repair assay, and increases formation of DNA damage-induced RAD51-positive foci. Importantly, gene expression in breast tumors supports our finding that E2F1 oppositely regulates *RAD51* and *TODRA*: increased *RAD51* expression, which is associated with an aggressive tumor phenotype (e.g. negative correlation with positive ER (r = -0.22, p = 0.02) and positive PR status (r = -0.27, p<0.001); positive correlation with ki67 status (r = 0.36, p = 0.005) and *HER2* amplification (r = 0.41, p = 0.001)), correlates as expected with lower *TODRA* and higher *E2F1* expression. However, although E2F1 induction resulted in *TPIP* downregulation in cell lines, we find that *TPIP* expression in tumors is not reduced despite higher *E2F1* expression, perhaps contributing to increased *RAD51* expression. Our results identify TPIP as a novel E2F1 co-activator, suggest a similar role for other TPTEs, and indicate that the *TODRA* lncRNA affects RAD51 dysregulation and RAD51-dependent DSB repair in malignancy. Importantly, gene expression in breast tumors supports our finding that E2F1 oppositely regulates RAD51 and TODRA: increased RAD51 expression, which is associated with an aggressive tumor phenotype (e.g. negative correlation with positive ER (r = -0.22, p = 0.02) and positive PR status (r = -0.27, p<0.001); positive correlation with ki67 status (r = 0.36, p = 0.005) and HER2 amplification (r = 0.41, p = 0.001)), correlates as expected with lower TODRA and higher E2F1 expression. However, although E2F1 induction resulted in TPIP downregulation in cell lines, we find that TPIP expression in tumors is not reduced despite higher E2F1 expression, perhaps contributing to increased RAD51 expression. Our results identify TPIP as a novel E2F1 co-activator, suggest a similar role for other TPTEs, and indicate that the TODRA lncRNA affects RAD51 dysregulation and RAD51-dependent DSB repair in malignancy.

## Introduction

RAD51 is the central recombinase involved in homologous recombination (HR), a mechanism for high fidelity repair of double-strand breaks (DSBs) that requires an intact, homologous DNA template[1]. DSBs are potentially lethal DNA lesions[2, 3], and disruption of their repair can lead to genomic instability, which plays an important role in both tumor initiation and progression[4]. Indeed, *RAD51* expression is often dysregulated in human tumors[5–7], promoting genomic instability[8, 9].

*RAD51* is regulated by members of the E2F transcription factor family, which plays a critical role in cell cycle control. E2Fs regulate expression of genes required for cell cycle-progression, DNA replication, mitosis, DNA damage response, checkpoint activation, differentiation, development, apoptosis and autophagy[10–14]. The E2F family is broadly subdivided into 'activator' E2Fs (E2F1-3a) and 'repressor' E2Fs (E2F4-8), based on their predominant effect on target gene expression. Both activator and repressor E2Fs bind the same recognition site in the *RAD51* promoter[15–19], and were shown to regulate *RAD51* expression during growth stimulation[17], hypoxia[18] and inhibition of poly(ADP-ribose) polymerase, a DNA-repair enzyme[19].

The *RAD51* locus contains an annotated long non-coding RNA (lncRNA) transcribed only 69bp upstream of *RAD51* in the opposite direction. We investigated whether this lncRNA, dubbed *TODRA*, regulates *RAD51* expression and activity. Our results suggest that *TODRA* participates in regulation of *RAD51* expression through E2F1 and TPIP, a member of the PTEN phosphatase family. To assess the functional effects of this regulatory mechanism we analyzed *RAD51*-dependent DSB repair as well as formation of RAD51-positive foci following DNA damage. We found that *TODRA* overexpression induces DSB repair by HR and also increases the fraction of RAD51 foci formed following DNA damage. In breast tumors, expression analysis of *RAD51*/*TODRA*, *E2F1* and *TPIP* shows perturbed regulation of *RAD51* expression, and the associated increase in *RAD51* expression correlates with an aggressive tumor phenotype.

## Results

### *AK125393 (TODRA)–*a lncRNA immediately upstream of *RAD51* is transcribed in the opposite direction

**Fig 1:**
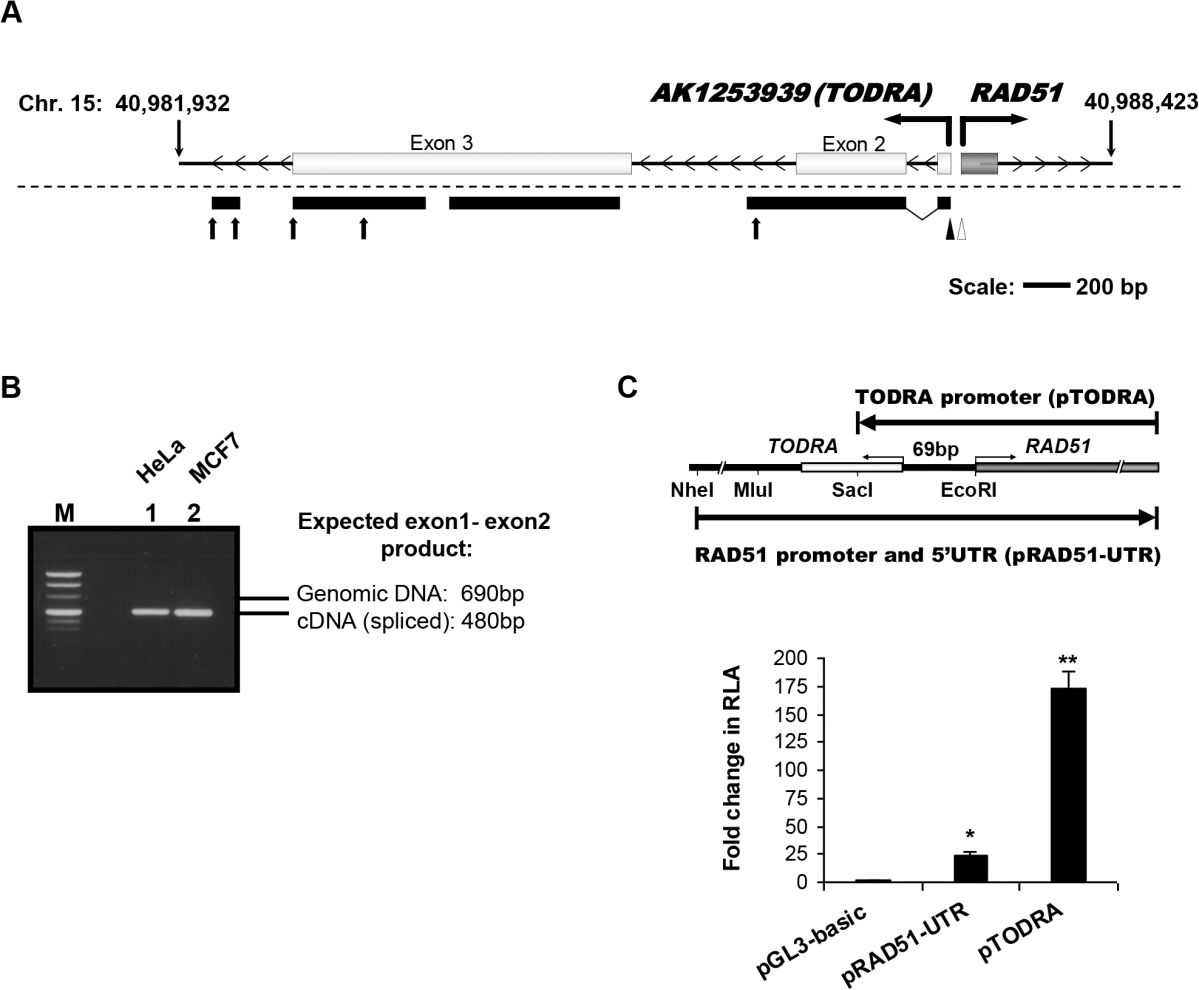
Transcriptional analysis of the *RAD51/TODRA* region. **A.** *TODRA* transcript: **Top:** Schematic representation of the predicted *TODRA* (*AK125393*) gene, as described in the UCSC genome browser. Light grey shaded rectangles depict *TODRA* exons, the dark grey rectangle depicts *RAD51* exon 1, transcribed in the opposite direction. **Bottom:** Results of *TODRA* transcript analysis. 5’RACE using capped HeLa mRNA, identified one transcription start site (full arrowhead, +1 corresponds to chr. 15: 40987374, hg19), and 3’RACE identified several possible transcription termini (arrows). The most 5’ end of *RAD51* identified using 5’RACE is also shown (empty arrowhead, +1 corresponds to chr. 15: 40987303, hg19). Black bars beneath the diagram indicate confirmed regions of unidirectional transcription determined using strand specific primers for reverse transcription from both HeLa and MCF7 cells. **B.** Splicing of *TODRA* exons 1 and 2 is demonstrated in the representative gel. Lane M: pUC Mix Marker, (Fermentas), Lanes 1&2: *TODRA* strand specific RT-PCR products (F primer located in exon 1, R primer in exon 2). Expected size of product in genomic DNA: 696bp, Expected size of spliced transcript: 480bp, as observed in lanes 1 (cDNA prepared from HeLa cells) and 2 (cDNA prepared from MCF7 cells). **C.** The *RAD51/TODRA* region supports transcription in both directions. **Top:** Schematic representation of the *RAD51* and *TODRA* promoter regions and the fragments cloned into luciferase promoter constructs. **Bottom:** *TODRA* putative promoter activity. MCF7 cells were co-transfected with the promoter-less pGL3-basic, pRAD51-UTR or pTODRA and pRL-TK (to normalize for transfection efficiency). Results are shown as fold increase in RLA (relative luciferase activity), compared to pGL3-basic. Values are means ± SE of 4–5 independent transfections performed in duplicates. * p< 0.002, ** p< 0.0001.

*AK125393* is a putative long non-coding RNA in the *RAD51* locus, identified through the FLJ (“full-length long Japan”) effort[20] to sequence full-length human cDNAs. *AK125393* lacks significant open reading frames (all <100aa), and was therefore annotated as a non-coding RNA (ncRNA). In the UCSC Genome Browser (assembly Feb. 2009 [GRCh37/Hg19]) *AK125393* is shown as a 3-exon non-coding expressed gene transcribed in the opposite direction to *RAD51 *(Fig 1A), and the transcription start sites (TSSs) of *RAD51* and *AK125393* are only 69bp apart. We named the *AK125393* ncRNA *TODRA*, for Transcribed in the Opposite Direction of *RA* *D51*. To confirm *TODRA* expression and its TSS, we performed strand specific RT-PCR and 5' and 3'RACE on cDNA. We demonstrated expression of *TODRA* exons 1–3, (as characterized in the UCSC and NCBI databases) (Fig 1A), and observed splicing of intron 1, indicating this RNA is indeed transcribed and processed (Fig 1B). Other transcription products were also observed, including previously described introns (specifically intron 2) and downstream 3' sequences (Fig 1A). Northern blot analysis using a number of probes did not identify a major transcription product.

To determine if the *RAD51* promoter region also supports transcription in the *TODRA* direction, this intergenic promoter region was cloned upstream of the firefly luciferase gene in the promoter-less pGL3-basic vector, in both forward (*RAD51*) and reverse (*TODRA*) orientations. These plasmids, respectively designated pRAD51-UTR and pTODRA (Fig 1C), were transfected into MCF7 cells and assayed for relative promoter activity. Both constructs drove transcription. In this assay, even though the *RAD51* construct is larger, and includes all known transcription promoting regions (pRAD51-UTR contains a larger insert in the *RAD51* direction, including *RAD51* promoter elements located both upstream to *TODRA*'s TSS and in the *RAD51* 5'UTR), pTODRA activity was 7-fold greater (p<0.0001) than that of pRAD51-UTR (Fig 1C), demonstrating that this region supports bidirectional transcription.

### The intergenic E2F site oppositely regulates *RAD51* and *TODRA* transcription

**Fig 2:**
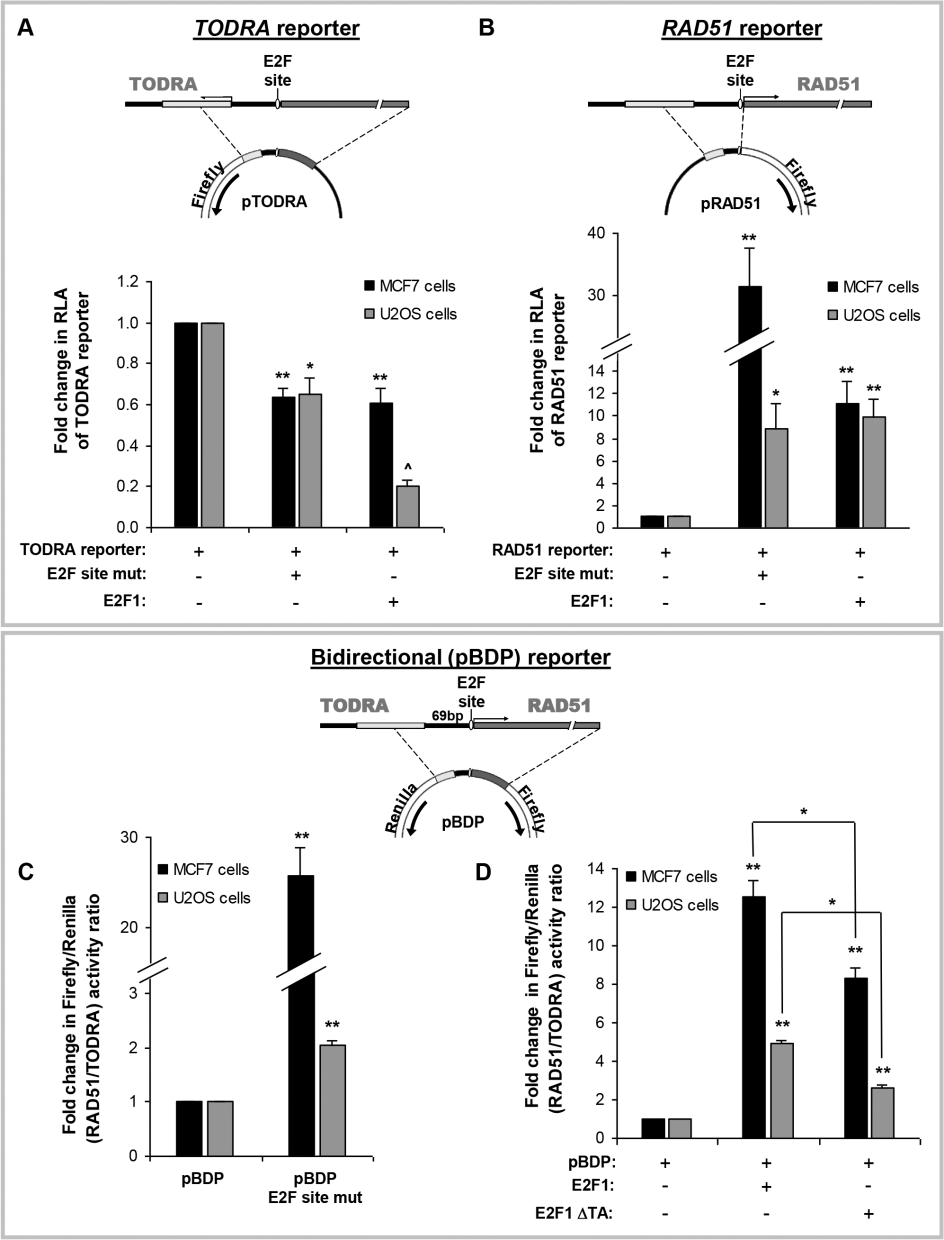
The E2F site in the common promoter region has opposite effects on *RAD51* and *TODRA*. **A. Top:** Diagram of the core *TODRA* promoter region cloned into the luciferase reporter vector. **Bottom:** Effect of mutagenesis of the E2F binding site and E2F1 induction on the *TODRA* reporter. Wild type *TODRA* luciferase (reporter) construct, or an E2F binding site mutant (E2F site mut) construct were transfected into MCF7 and U2OS cells. A *TODRA* luciferase (reporter) construct was also co-transfected with either an E2F1 expression vector or an empty vector control into serum-starved MCF7 and U2OS cells. All experiments included co-transfection with pRL-TK (to normalize for transfection efficiency). Results are depicted as the fold change in RLA compared to the WT construct transfection. Values in all experiments are means ± SE of 3–4 independent transfections performed in duplicate. ** p≤ 0.007, * p≤ 0.02, ^ p = 0.00001. **B. Top:** Diagram of the core *RAD51* promoter region cloned into the luciferase reporter vector. **Bottom:** Effect of mutagenesis of the E2F binding site and E2F1 induction on the *RAD51* reporter. Wild type *RAD51* luciferase (reporter) construct, or an E2F binding site mutant (E2F site mut) construct were transfected into MCF7 and U2OS cells. A *RAD51* luciferase (reporter) construct was also co-transfected with either an E2F1 expression vector or an empty vector control into serum-starved MCF7 and U2OS cells. All experiments included co-transfection with pRL-TK (to normalize for transfection efficiency). Results are depicted as the fold change in RLA compared to the WT construct transfection. Values in all experiments are means ± SE of 3–4 independent transfections performed in duplicate. ** p≤ 0.007, * p≤ 0.02. **C. and D. Top:** Diagram of the *RAD51/TODRA* bidirectional promoter region cloned between the firefly and Renilla luciferase reporter genes (pBDP). **C.** Mutagenesis of the E2F binding site. E2F site mutant (pBDP E2F site mut) or wild type bidirectional promoter constructs (pBDP) were transfected into MCF7 and U2OS cells. Results are depicted as the fold change in the mutant compared to the WT in the ratio of Firefly/Renilla luciferase activities, which represents the ratio of *RAD51/TODRA* promoter activities. Values are means ± SE of 3–6 independent transfections performed in duplicate. ** p< 0.0001. **D.** E2F1 overexpression. pBDP activity was examined in MCF7 and U2OS cells co-transfected with the pBDP construct and either an E2F1 WT, an E2F1 trans-activation domain deletion mutant (ΔTA), or an empty expression vector. Results are depicted as the fold change between each E2F1 expression vector and the empty vector control, in the ratio of Firefly/Renilla luciferase activities, which represents the ratio of *RAD51/TODRA* promoter activities. Values are means ± SE of 3–6 independent transfections performed in duplicate. ** p< 0.0001. Additional comparisons are indicated above the bars. * p≤ 0.003.

Both E2F1 and E2F4 have previously been shown to bind to the *RAD51* proximal promoter and regulate *RAD51* expression[17–19]. While E2F1 activates expression[17], E2F4/p130 complexes repress *RAD51* [18, 19], through the E2F1/E2F4 binding site located within the shared *RAD51/TODRA* promoter. To examine its role in *TODRA* expression, this E2F binding site was mutated based on mutations previously show to abolish the binding site[17] (TTTGGCGGGAAT→TT**C**GG**AC**GGAAT) and assayed for the effect on promoter activity. In both MCF7 and U2OS cells, abolishing the E2F binding site repressed pTODRA activity by approximately 1.5-fold (p<0.01) (Fig 2A). In contrast, activity of pRAD51, the minimal *RAD51* promoter construct, was increased by 30-fold (p<0.003) in MCF7 cells and ~9-fold (p = 0.02) in U2OS cells (Fig 2B). This is consistent with previous studies of *RAD51* in MCF7 cells, which showed that under baseline conditions the *RAD51*-E2F site is occupied mainly by the repressive E2F4 factor[18]. Mutagenesis of the E2F site therefore prevents E2F4 binding and de-represses *RAD51*. Overexpression of E2F4 did not affect either *RAD51* or *TODRA* promoter activities (S1 Fig). However, E2F1 overexpression resulted in a 10-11-fold increase (p≤0.005) of *RAD51* promoter activity (pRAD51-UTR) (Fig 2B), and a 40–80% reduction (p≤0.007) in *TODRA* promoter activity (Fig 2A). To summarize, our results show that *TODRA* is indeed regulated by the shared E2F binding site, and that E2F1 has an opposite effect on *RAD51 vs*. *TODRA* expression.

The effect of the E2F site was further analyzed using a bidirectional promoter (BDP) construct mimicking the endogenous bidirectional promoter region. A dual reporter vector was constructed by inserting the overlapping promoter region (analogous to the pTODRA construct) between firefly and Renilla luciferase genes oriented in opposite directions (Fig 2C and 2D). In this pBDP construct, changes in the ratio of Firefly to Renilla luciferase activities represent changes in the ratio of *RAD51/TODRA* promoter activities. Mutagenesis of the E2F site increased the Firefly/Renilla ratio by 25-fold (p<0.0001) in MCF7 cells and 2-fold (p = 0.001) in U2OS cells, corresponding to an increased ratio of *RAD51/TODRA* promoter activities (Fig 2C). This is consistent with results from the unidirectional E2F site mutagenized promoter constructs (Fig 2A and 2B). E2F1 overexpression resulted in 12-fold and 5-fold (p≤0.00002) increase in the Firefly/Renilla activity ratios, in MCF7 and U2OS cells respectively, also corresponding to an increased *RAD51/TODRA* expression ratio (Fig 2D). Overexpression of an E2F1 transactivation domain mutant (E2F1ΔTA) resulted in a milder, 8-fold and 2.6-fold (p<0.0001) induction in MCF7 and U2OS cells respectively (Fig 2D). This suggests that even just the binding of E2F1 to its site is sufficient to modulate the *RAD51/TODRA* expression ratio. Taken together, these results indicate that in the bidirectional promoter, transcription factor binding to the E2F site results in differential expression of the *RAD51* and *TODRA* genes, and that E2F1, a known *RAD51* activator, acts simultaneously as a transcriptional repressor of *TODRA*. This is only partly explained by E2F1-induced transactivation.

### E2F1 regulates endogenous *RAD51* and *TODRA* transcript levels

**Fig 3:**
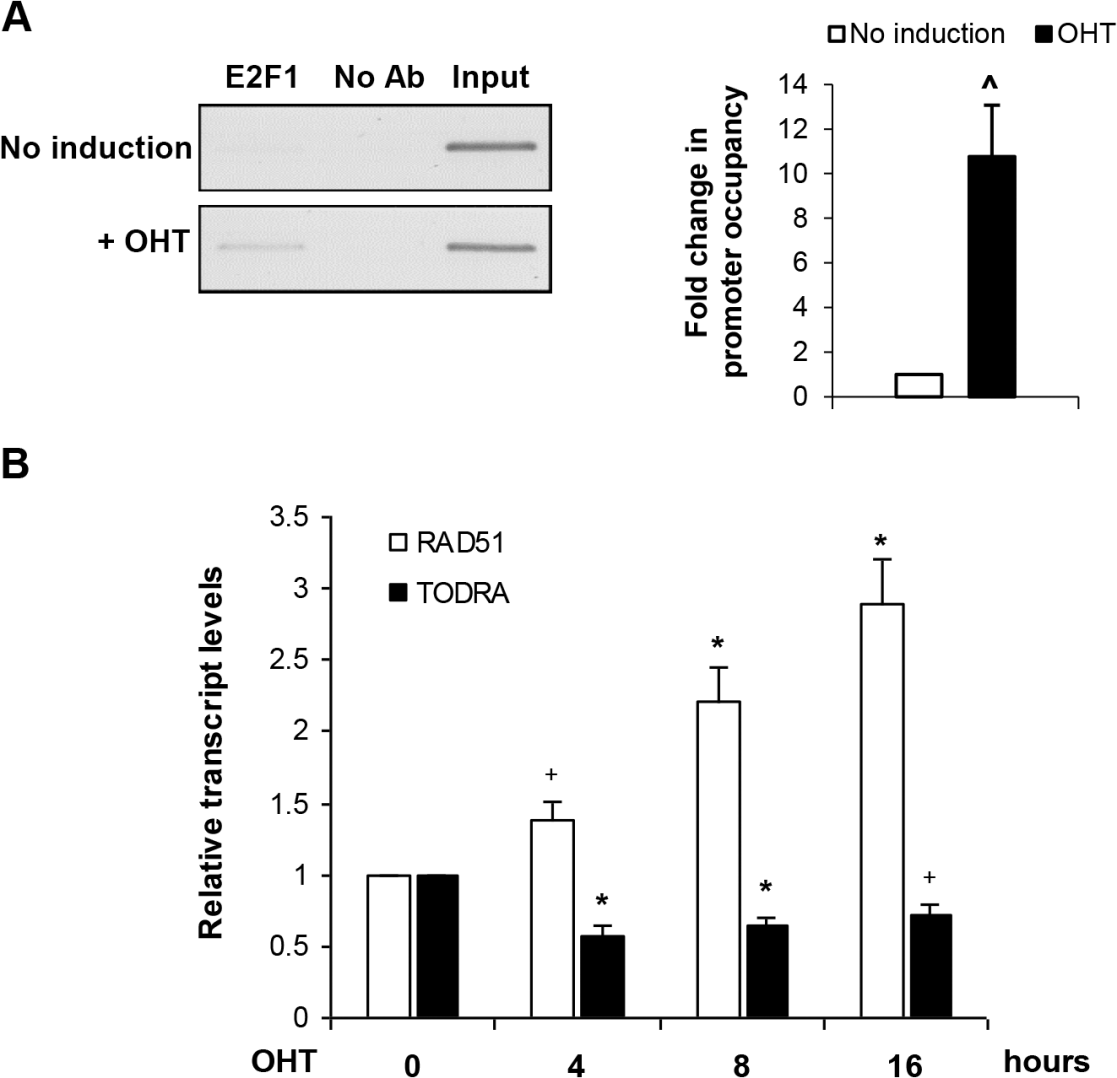
E2F1 induction oppositely affects endogenous *RAD51* and *TODRA* expression. **A.** E2F1 induction results in E2F1 binding of the *RAD51/TODRA* promoter. E2F1 expression was induced in serum starved ER-E2F1 U2OS cells (stably transfected with a constitutively expressed ER-E2F1 fusion protein which upon ligand-dependent activation translocates from the cytoplasm to the nucleus) by treatment with OHT for 8 hours. *RAD51/TODRA* promoter occupancy was measured with a ChIP assay using E2F1 antibodies (Ab) in lysates of either OHT treated or untreated cells. Real-time PCR was performed to quantitate the *RAD51/TODRA* template captured by the E2F1 Ab. Promoter occupancy is expressed as fold change relative to that observed in untreated cells. Values are means ± SE of 3 ChIP independent experiments. Real-time reactions were performed in triplicates. ^ p = 0.01. A representative gel of the promoter region PCR amplification products is shown on the left (S2 Fig). **B.** E2F1 induction and endogenous *RAD51* and *TODRA* transcription. *RAD51* and *TODRA* transcript levels were determined after E2F1 induction (see above), using quantitative real-time RT-PCR normalized to *GAPDH*. Results are depicted as the fold change in either *RAD51* or *TODRA* transcript levels compared to non-treated cells (time 0). Values are means ± SE of 3–4 independent experiments. Real-time reactions were performed in duplicates. + p≤ 0.04, * p≤ 0.004.

To determine the effect of E2F1 on activity of the endogenous *RAD51/TODRA* promoter we utilized a U2OS cell line stably transfected with a conditionally active E2F1 construct (ER-E2F1), in which 4-OHT (4-hydroxytamoxifen) treatment induces E2F1[21]. Elevated E2F1 occupancy of the *RAD51* promoter after OHT treatment was confirmed using chromatin immunoprecipitation (ChIP) (Fig 3A). E2F1 induction resulted in a 3-fold increase (p = 0.004) in endogenous *RAD51* mRNA levels and a 40% decrease (p = 0.0005) in endogenous *TODRA* transcript levels (Fig 3B). These results demonstrate that E2F1 oppositely regulates the endogenous *RAD51/TODRA* bidirectional promoter, providing *in vivo* evidence for its role in regulating both *RAD51* and *TODRA* expression.

### *TODRA* promotes both RAD51-dependent homologous recombination (HR) repair of an *ISce*I-induced DSB, and formation of DNA damage-induced RAD51 foci.

**Fig 4:**
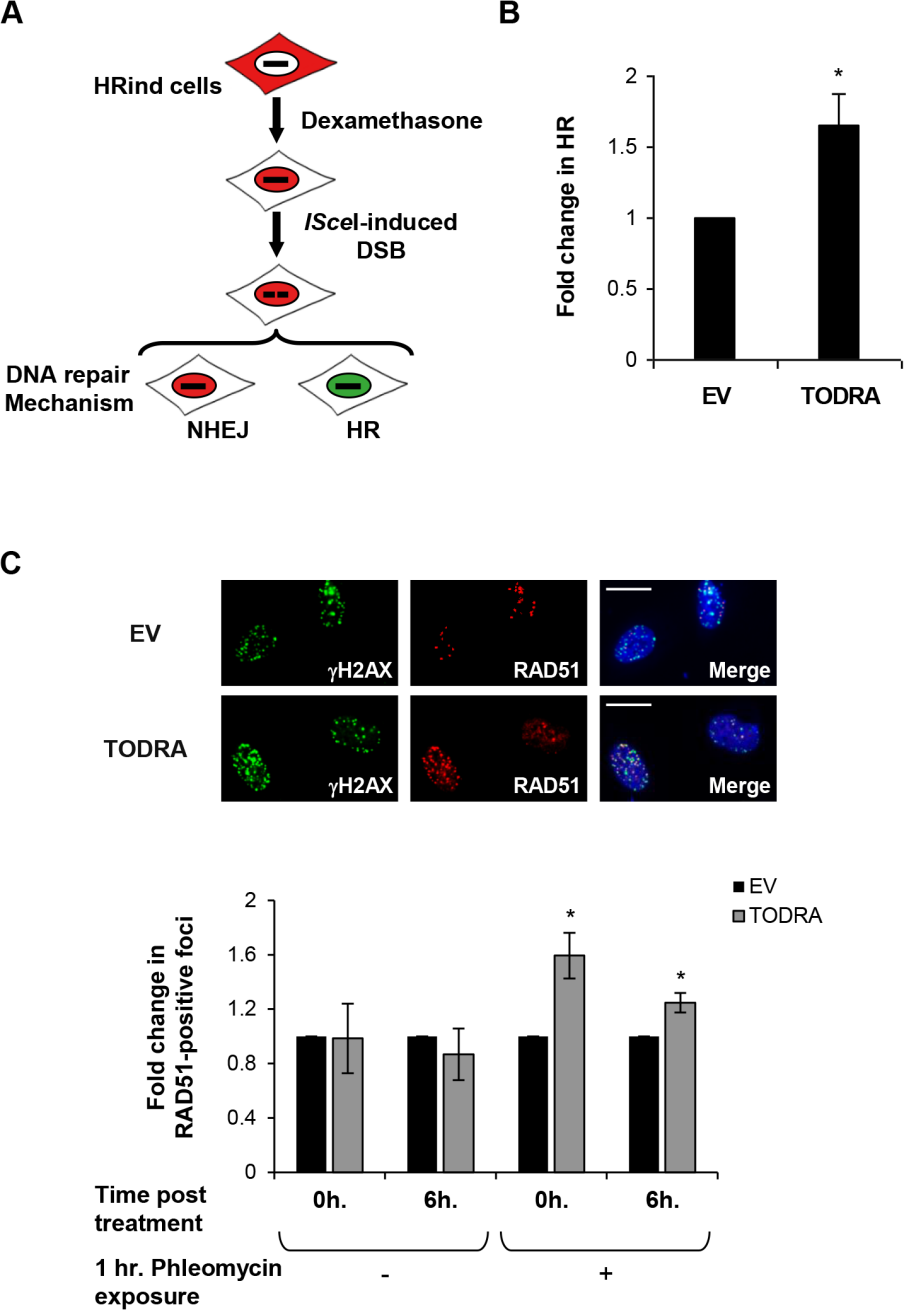
*TODRA* promotes homologous recombination repair of DSBs. **A.** Schematic representation of the HRind cell system **.** The mCherry-*ISce*I-GR (Glucoroticoid Receptor) endonuclease is cytoplasmic. Upon addition of Dexamethasone, it rapidly translocates into the nucleus generating a DSB at the *ISce*I site in the DR-GFP cassette. The DSB can be repaired either by NHEJ (non-homologous end-joining) or HR, but only HR repair reconstitutes functional GFP (green nucleus) from DR-GFP. **B.** Overexpression of *TODRA* induces RAD51-dependent HR. HRind cells were transfected with an empty vector (EV) or *TODRA* minigene and induced with Dexamethasone for 48 hours. GFP expression was measured by FACS. Results are depicted as the fold change in observed HR (as indicated by the number of GFP-positive cells) compared to the empty vector. Values are means ± SE of 3 independent experiments performed in triplicate. * p< 0.04. **C.** Overepxression of *TODRA* elevates DNA damage-induced RAD51 foci formation. U2OS cells were transfected with an empty vector (EV) or the *TODRA* minigene. 48 hrs. post transfection half of each culture was treated for 1 hr. with the DNA damaging agent phleomycin (10μg/ml). Medium was then replaced in all cultures, releasing treated cells from phleomycin exposure. γH2AX and RAD51 foci were imaged either immediately (0 hours) or 6 hours after removal of phleomycin and medium exchange. **Top:** A representative image of γH2AX (green) and RAD51 (red) foci in empty vector (EV) and *TODRA* transfected cells 6 hours after removal of phleomycin. DAPI (blue signal in merged images) was used for counterstaining. Scale bars = 10 μm. **Bottom:** The number of RAD51-positive foci was normalized as the fraction of γH2AX-positive foci per cell and averaged across all samples in each condition. Cells were treated with phleomycin, as indicated, and fixed at the indicated time points post-treatment. Results are depicted as the fold change in the fraction of RAD51 foci in cells overexpressing *TODRA* compared to the empty vector. Values are means ± SE of 3 independent experiments. * p≤ 0.03.

To determine the functional relevance of *TODRA* to RAD51's role in DSB repair, we used a RAD51-dependent DSB repair assay and examined formation of RAD51 positive foci following DNA damage. To assess its effect on DSB repair, we expressed *TODRA* in HR-inducible (HRind) U2OS cells stably transfected with both a DR-GFP HR reporter cassette and an mCherry-*ISce*I-GR (Glucocorticoid Receptor) plasmid[22]. Addition of Dexamethasone to the cell media, translocates the *ISce*I endonuclease to the nucleus, where it generates a unique DSB within the DR-GFP cassette. Functional GFP is produced only upon HR repair of the DSB (Fig 4A). *TODRA* overexpression significantly increased the number of GFP-positive cells, by 1.6 fold (p<0.04) (Fig 4B). This change is of comparable magnitude to that seen with *RAD51* depletion (which decreases HR by ~2.5-fold)[23].

In addition, we analyzed RAD51 foci formation in U2OS cells treated with the DNA-damaging agent phleomycin. Overexpression of *TODRA* increased the proportion of RAD51-positive foci by 1.25–1.6 fold in transfected cells (Fig 4C) (p<0.03 vs. empty vector). Thus, *TODRA* overexpression resulted in similar increases of RAD 51-dependent repair activity using two different assays.

### TPIP, a potential *TODRA* target gene, induces *RAD51* expression synergistically with E2F1

To identify genes that may be regulated by *TODRA* and thus influence RAD51-dependent HR, we attempted to knock-down endogenous *TODRA* expression. However, we were unable to attain effective *TODRA* knockdown (we achieved less than 50% reduction in transcript levels). We therefore used the Affymetrix GeneChip Human Gene 1.0 ST expression array to screen for genes affected by overexpression of a *TODRA* minigene in HeLa cells. We observed no significant change in *RAD51* mRNA levels; however, the *TPIP* transcript was significantly upregulated by *TODRA* overexpression.

**Fig 5:**
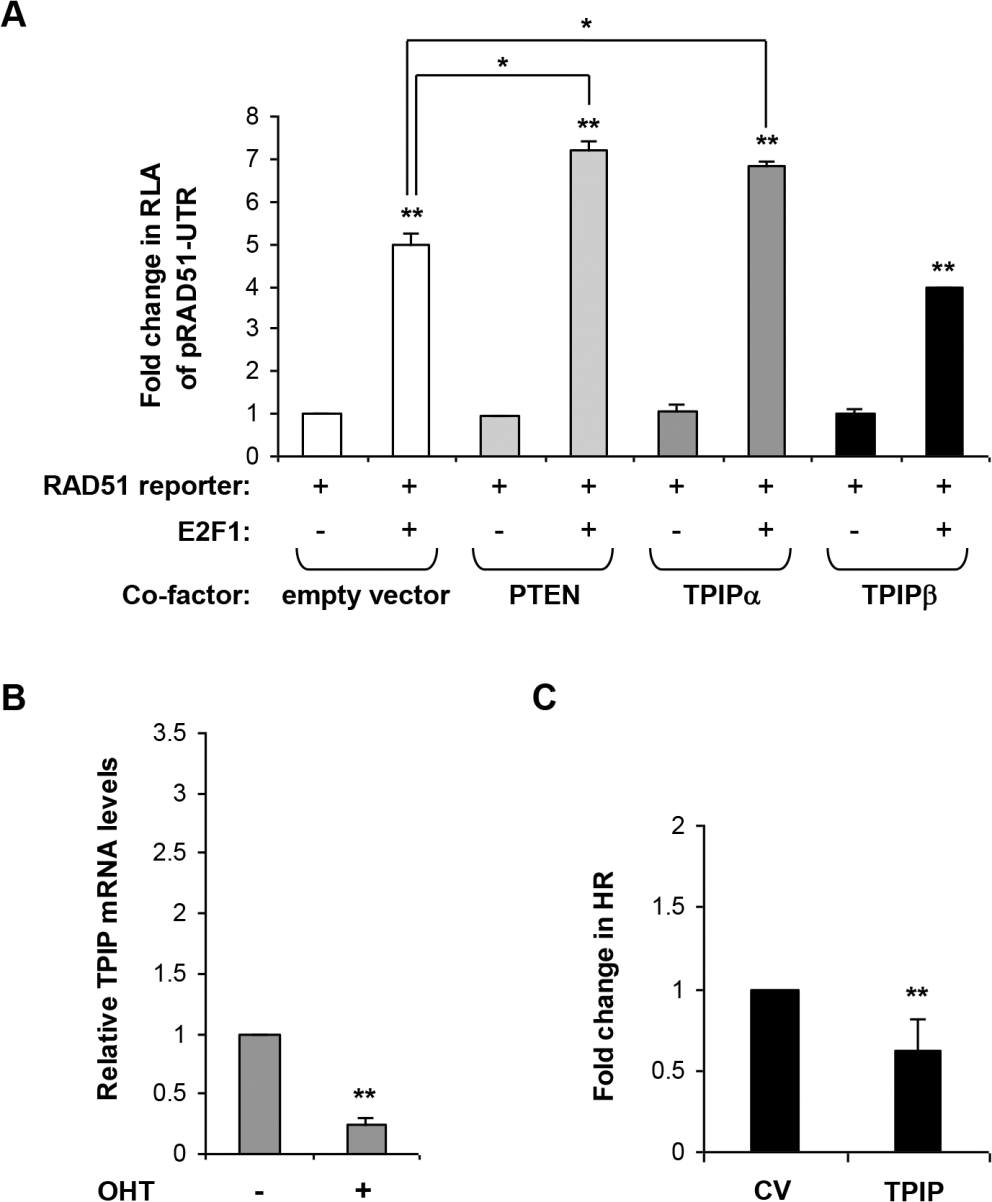
TPIP regulates *RAD51* expression and activity. **A.** TPIP co-activates E2F1 induction of *RAD51*. pRAD51-UTR with an E2F1 expression vector or an empty vector were co-transfected into serum starved MCF7 cells together with either PTEN, TPIPα, TPIPβ or an empty pEGFP-C2 based expression vector and pRL-TK (to normalize for transfection efficiency). Results are depicted as fold change in RLA compared to pRAD51-UTR alone (left bar). Values are means ± SE of 3 independent transfections performed in duplicates. ** p< 0.0001. Additional comparisons are indicated above the bars. * p = 0.002. **B.** E2F1 induction and endogenous *TPIP* expression. Endogenous *TPIP* mRNA levels were determined using quantitative real-time RT-PCR normalized to *GAPDH*, with and without E2F1 induction in serum starved ER-E2F1 U2OS cells. E2F1 was induced by treatment with OHT for 4 hours. Values are means ± SE of 4 independent experiments. Real-time reactions were performed in duplicates. ** p< 0.00001. **C.** Overexpression of *TPIP* reduces HR. HRind cells were transfected with an mOrange2 control vector (CV) or *TPIP* expression vector (tagged with mOrange2) and induced with Dexamethasone for 48 hours. GFP expression was measured by FACS. Results are depicted as the fold change in observed HR (as indicated by the number of GFP-positive cells among the transfected population [mOrange2 positive cells]) compared to the control vector. Values are means ± SE of 3 independent experiments performed in triplicate. ** p<0.002.

*TPIP* belongs to the human *TPTE* (Transmembrane Phosphatase with TEnsin homology) gene family which encodes PTEN-related tyrosine phosphatases[24, 25]. PTEN was shown to transactivate the *RAD51* promoter synergistically with E2F1, a member of the E2F transcription family[26]. We therefore investigated whether TPIP affects *RAD51* promoter activity. TPIPα TPIPβ (predominant TPIP isoforms) or PTEN expression vectors[25] were co-transfected into MCF7 cells with a *RAD51* promoter luciferase reporter (pRAD51-UTR) together with an E2F1 expression vector. Like PTEN, TPIP alone did not activate the *RAD51* promoter. However, when co-expressed with E2F1, TPIPα (but not TPIPβ) augmented E2F1 induction of *RAD51* promoter activity (by 1.4-fold (p = 0.002)), as compared to E2F1 alone (Fig 5A). These results demonstrate that like PTEN, TPIP acts as a co-factor of E2F1, inducing *RAD51* expression.

To further investigate the interaction between *TPIP* and E2F1, we examined whether E2F1 can also regulate *TPIP* expression. Interestingly, much like for *TODRA* (Fig 3B), E2F1 overexpression resulted in a 4-fold decrease (p<0.00001) in *TPIP* mRNA levels (Fig 5B). This suggests that *TPIP* transcription is regulated in an E2F1-dependent manner, in parallel to *TODRA*.

Given that *TPIP* expression is modified by *TODRA*, and that the TPIP protein co-regulates *RAD51* expression, we next asked whether *TODRA*'s effect on RAD51-dependent HR is achieved via TPIP. We overexpressed *TPIP* in HR-inducible (HRind) U2OS cells described above and analyzed GFP expression in the transfected cells. *TPIP* overexpression reduced the number of GFP-positive cells, by 1.6 fold (p = 0.001) (Fig 5C). This suggests that *TODRA* increases RAD51-dependent HR independently of TPIP.

### The *RAD51/TODRA* pathway in breast cancer

We also examined the *RAD51-TODRA* pathway *in vivo*, in breast tumors. Because *TODRA* has been described only recently, it is not represented in expression arrays used in many studies (e.g. the often cited van’t Veer *et al*. study[27]). We therefore analyzed data obtained by Muggerud *et al*.[28] who studied global gene expression of 109 breast tumors and 6 normal breast tissues using Agilent Whole Human Genome Oligo Microarrays 44k, which contain probes for *RAD51*, *E2F1*, *TODRA* and *TPIP*. Correlation of these genes' expression (Table 1) shows a negative correlation between *RAD51* and *TODRA* (p = 0.01), reflecting their opposite regulation by E2F1, which is positively correlated with *RAD51* (p< 0.001) and negatively with *TODRA* (p = 0.002). However, in contrast to our findings in U2OS cells (Fig 5B), *E2F1* expression was positively correlated with *TPIP* expression, and there was no correlation between *TODRA* and *TPIP* expression. High expression of *RAD51* and *E2F1* also correlated with clinico-pathological features, and was associated with young age at onset (p = 0.002), a trend toward lymph node involvement at diagnosis (p = 0.08) and markers of aggressive disease, i.e. high tumor grade, lack of estrogen and progesterone receptor expression, Her2 amplification and ki67 staining (Table 1). *TODRA* expression was not significantly correlated with clinico-pathological features, but in general its effect was opposite to that of *RAD51*.

**Table 1:** The *RAD51-TODRA* regulatory pathway in breast cancer tumors.

**Correlation of gene-gene expression**				
**Transcript 1**	**Transcript 2**	**Correlation direction**	**correlation coefficient (*r*)**	***p*-value**
*RAD51*	*TODRA*	-	0.24	0.01
*E2F1*	*RAD51*	+	0.67	<0.001
*E2F1*	*TODRA*	-	0.30	0.002
*E2F1* *	*TPIP* *	*+*	*0*.*22*	*0*.*02*
*TPIP*	*RAD51*	+	0.16	0.09 (NS)
*TODRA* *	*TPIP* *	*None*	*-*	*-*
**Correlation of gene expression and clinico-pathological features**				
**Transcript**	**Clinico-pathological feature**	**Correlation direction**	**correlation coefficient (r)**	**p-value**
	Age at diagnosis	-	0.30	0.002
	Lymph node involvement at diagnosis	+	0.19	0.08 (NS)
*RAD51*	Tumor grade	+	0.43	<0.001
	Positive ER status	-	0.22	0.02
	Positive PR status	-	0.27	<0.001
	HER2 amplification	+	0.41	0.001
	ki67 status	+	0.36	0.005
	Age at diagnosis	-	0.25	0.008
	Lymph node involvement at diagnosis	+	0.21	0.04
*E2F1*	Tumor grade	+	0.37	<0.001
	Positive ER status	-	0.26	0.03
	Positive PR status	-	0.22	0.001
	HER2 amplification	+	0.22	0.03
	ki67 status	+	0.34	0.002
	Tumor grade	+	0.20	0.04
*TPIP*	Positive ER status	-	0.30	0.01
	Positive PR status	-	0.34	0.004

Relationship between transcript expression levels, along the *RAD51-TODRA* regulatory pathway, in breast cancer tumors (based on data from Muggerud *et al*., 2010[28]). + positive correlation,—negative correlation. NS: not significant. All p-values are for 2-tailed analysis. Pearson correlation was used for comparison of continuous variables and Spearman correlation and t-test for non-parametric comparisons.

* Asterisks indicate gene-gene correlations that reflect perturbation of the normal pathway.

## Discussion

RAD51 is an essential recombinase, often dysregulated in tumors[5–7], and tight regulation of its expression is pivotal for sustaining genome stability. We hypothesized that a novel lncRNA (*AK125393*) at the *RAD51* locus, transcribed in the opposite direction of RA*D5*1 (namely, *TODRA*) may play a role in *RAD51* regulation. This hypothesis was based on the emerging roles of ncRNAs in general and lncRNAs in particular[29–31]. LncRNAs share features with protein coding genes: they are frequently long (generally >2kb, and some >100kb), spliced and contain canonical polyadenylation signals. In addition, lncRNA promoters are bound and regulated by transcription factors, e.g. Oct3/4, CREB, and p53, and epigenetically marked with specific histone modifications[32]. We found that *TODRA's* transcription indeed initiates 69bp upstream of the *RAD51* TSS, and confirmed that it is transcribed and spliced using strand-specific RT-PCR (Fig 1A and 1B). Northern blotting did not identify a major transcription product, perhaps due to instability or heterogeneity of the transcript, consistent with evidence that lncRNAs are often degraded or processed into small RNAs[33–36]. We determined that the *RAD51* promoter region supports bidirectional transcription, acting as a strong promoter in the direction of *TODRA* as well as in the direction of *RAD51* (Fig 1C). Thus, *TODRA* is a *bona fide* lncRNA which is driven by the bidirectional *RAD51* locus promoter.

Bidirectional promoters are a common organizational motif in the human genome, and notably, a number of DNA-repair genes are arranged in bidirectional pairs separated by less than 300bp[37, 38]. Most bidirectional promoters involving DNA-repair genes have not been thoroughly investigated, but two studied examples include the *BRCA1/NRB2* locus[39–41] and the *ATM/NPAT* promoter[42]. Similar to *RAD51* and *TODRA*, *BRCA1* and *NBR2* (neighbour of *BRCA1* gene 2) are aligned in a head-to-head orientation separated by 218bp, and *NRB2*, which is annotated as a ncRNA, is transcribed in the opposite direction to *BRCA1*.

LncRNAs constitute a large portion of the mammalian transcriptome, and play a regulatory role in a range of biological pathways[29–31]. Impaired lncRNA expression and function contribute to disease pathogenesis, including cancer[32, 43–45]. For example, *MEG3* (maternally expressed gene 3), is an imprinted, maternally expressed gene, encoding a lncRNA that functions as a tumor suppressor. *MEG3* regulates TP53 protein levels and plays a role in tumor cell proliferation, apoptosis and angiogenesis. Its expression is lost in various human tumors (e.g. human pituitary tumors derived from gonadotroph cells and bladder cancer) as well as in tumor cell lines of various origins[46, 47]. Moreover, a more global pattern of altered lncRNA expression has been reported in both breast and ovarian cancers[44, 48].

LncRNA promoters, like protein coding genes, are regulated by transcription factors [32]. The *ATM/NPAT* bidirectional promoter noted above is regulated by E2F1, which induces expression of both genes[42]. Indeed, E2F1/E2F4 recognition sequences are significantly over-represented in bidirectional compared to unidirectional promoters[49]. The *RAD51/TODRA* promoter region also contains an E2F binding site that was previously studied in the context of *RAD51* regulation[17–19]. In quiescent and hypoxic cells this E2F site is primarily occupied by E2F4 (a component of the repressor complex E2F4/p130) whereas in response to growth stimulation, E2F4 is displaced and *RAD51* expression is activated by binding of E2F1 to the same E2F site[17, 18]. We found that the E2F site also regulates *TODRA* expression, but its effects are opposite to those on *RAD51*. Abolishing the E2F site, both in uni- and bi-directional constructs, increased *RAD51* promoter activity but decreased *TODRA* promoter activity (Fig 2). The E2F site can bind various members of the E2F family, but since E2F1 and RAD51 are often dysregulated in cancer, we focused on the effect of E2F1 on the bidirectional promoter. We found that while E2F1 overexpression induced *RAD51* promoter activity, as previously reported[17], it reduced *TODRA* promoter activity (Fig 2). *In vivo*, E2F1 induction increased endogenous *RAD51* mRNA levels and decreased endogenous *TODRA* transcript levels (Fig 3B). Deletion of the transactivating domain of E2F1 attenuated, but did not abolish, these effects (Fig 2D). This suggests that the E2F1 effect at the *RAD51/TODRA* promoter is mediated via two mechanisms: 1) E2F1 binding displaces E2F4, which is normally bound to this site, thereby de-repressing *RAD51* expression and 2) E2F1 actively induces the *RAD51* promoter and represses *TODRA* expression. This dual effect can explain why both mutagenesis of the E2F site and E2F1 overexpression result in *RAD51* activation and *TODRA* repression. It can also explain the partial effect of the E2F1 transactivating mutant, which can displace E2F4, but cannot act as a transcriptional activator. This model is also supported by previous studies which showed alternate occupancy of the *RAD51* promoter by E2F1 and E2F4[17, 18].

*RAD51* regulation by E2Fs conforms to the common functions of E2F1 as a transcriptional activator and E2F4 as a repressor[10]. However, as our results on *TODRA* and *TPIP* expression reveal, this dichotomy is not universal. E2F1 decreases expression of many genes, e.g., *BCL3*, *TGFB2* and *INHBA*[50, 51], and E2F4 can activate expression of numerous genes, including genes involved in cell cycle, DNA replication and DNA-repair (e.g. *RPA1* and *SMC3*)[52]. Taken together, our results demonstrate that an E2F site in the *RAD51/TODRA* bidirectional promoter differentially regulates *RAD51* and *TODRA* expression. To the best of our knowledge this is the first example showing opposing effects of E2F1 binding at a single site on transcription of two genes from a bidirectional promoter.

The functional relevance of *TODRA* with respect to RAD51 function was examined by analysis of RAD51 foci formation following DNA damage as well as a RAD51-dependent DSB repair assay (Fig 4). In these assays, *TODRA* significantly increased both the fraction of RAD51-positive DNA damage-induced foci and HR repair efficiency (Fig 4B and 4C). To further explore *TODRA*'s role in HR we explored potential targets that might mediate its activity. We found that *TODRA* overexpression upregulates *TPIP* (TPTE and PTEN homologous Inositol lipid Phosphatase, MIM #606791), providing a new example of a lncRNA regulating transcription of a protein-coding gene in another locus. *TPIP* encodes a PTEN-related tyrosine phosphatase, but unlike *PTEN*, little is known about *TPIP*'s function. *TPIP* is highly expressed in the testis and at lower levels in the brain and the stomach. Extensive alternative splicing of this gene leads to multiple isoforms. Two predominant forms, TPIPα and TPIPβ, differ at both the N- and C-termini, and only isoform α displays phosphoinositide 3-phosphatase activity[25]. PTEN has been shown to augment E2F1 induction of *RAD51*[26]. We found that TPIPα, but not TPIPβ, has a similar effect on *RAD51* expression (Fig 5A). This raises the possibility that other TPTEs also co-regulate *RAD51* expression.

Given our findings that *TODRA* enhances both *TPIP* expression and RAD51-dependent DNA repair, and that TPIP co-activates *RAD51*, the simplest hypothesis was that *TODRA* increases RAD51-dependent HR via upregulation of TPIP. However, this was not the case; Overexpression of *TPIP* decreased RAD51-dependent HR (Fig 5C). Even so, we found that both genes now newly implicated in regulating *RAD51* expression, affect RAD51-dependent HR. The opposite effects of *TODRA* and TPIP expression on this process probably reflect its complex regulation. RAD51-dependent double strand break repair is regulated on many non-transcriptional levels, including post-transcriptional modifications such as phosphorylation of both RAD51[53–56] and its partner, BRCA2[57]. Interestingly, a recent study[58] demonstrates that nuclear phosphoinositide signaling may play a role in nuclear export of *RAD51* mRNA. Thus, while TPIP can increase *RAD51* expression in certain settings (e.g. when E2F1 is expressed), it might also reduce its translation and functional activity by hampering the export of newly transcribed *RAD51* transcripts to the cytoplasm.

Finally, we examined the expression of genes associated with the *RAD51-TODRA* regulatory pathway in breast cancer tumors. *RAD51* expression in breast tumors was positively correlated with *E2F1* expression and negatively correlated with *TODRA* (Table 1), indicating that E2F1 indeed regulates the bidirectional promoter *in vivo* in the malignant state, in the same manner we observed in cell lines. Although in cell lines we found that E2F1 induction reduces *TPIP* expression, in breast tumors there was a positive correlation between *E2F1* and *TPIP* expression levels. This observation could indicate loss of normal fine-tuning of *RAD51* expression in the malignant *vs*. the normal state. While *RAD51* and *TODRA* expression are directly linked, through a shared E2F-binding site, the effect on *TPIP* may be less direct, and thus more likely to be affected by additional factors. E2F1 pathways are commonly dysregulated in cancer[10], and increased *TPIP* expression in the presence of increased *E2F1* expression in tumors could reflect dysregulation of *TPIP* transcription.

Importantly, the positive correlation between *E2F1/TPIP* and *RAD51* expression we show in tumors is consistent with our finding that TPIP is a novel co-activator of E2F1 in *RAD51* induction. Increased RAD51 levels may contribute to tumorigenesis, and accordingly we found that elevated *RAD51* levels are associated with young age at breast cancer onset, higher tumor grade and characteristics of aggressive tumors (e.g. lack of hormone receptor expression and *HER2* amplification).

**Fig 6:**
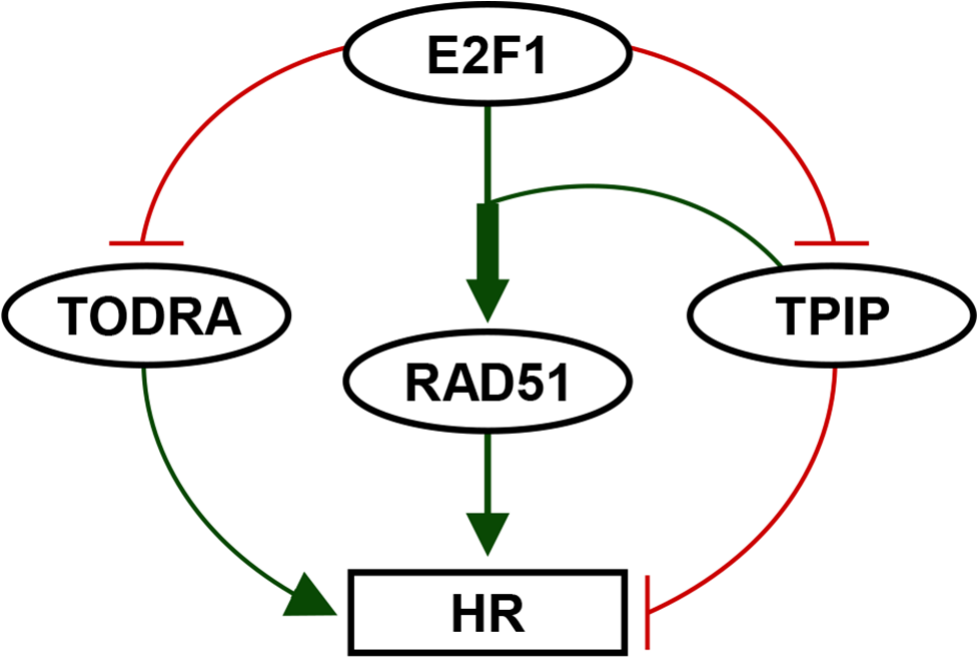
*TODRA* lncRNA plays a role in a new feedback loop regulating *RAD51* expression and activity. E2F1 induction enhances *RAD51* expression (thin green arrow) while simultaneously reducing lncRNA *TODRA* expression. While E2F1 induction of *RAD51* is synergistically enhanced by TPIP (thick green arrow), E2F1 induction also reduces *TPIP* expression, possibly by affecting *TODRA* expression, as *TODRA* expression can increase *TPIP* expression. This feedback regulation of *RAD51* expression can fine-tune *RAD51* expression and HR-DSB repair. Green: Enhancement of expression/activity. Red: Suppression of expression/activity.

To summarize, our findings reveal novel and complex regulatory mechanisms of *RAD51* expression and activity. We found that a lncRNA, *TODRA*, regulates both the expression and the activity of a protein coding gene (*RAD51*), driven, and oppositely regulated, by the same promoter. In a feedback loop (Fig 6), *TODRA* overexpression increases levels of *TPIP*, which we subsequently identified as a new *RAD51* co-activator. Additionally, both *TODRA* and TPIP are able to fine-tune homologous recombination, the primary activity of RAD51. Further investigation is needed to determine the mechanism that underlies *TODRA* regulation of *TPIP* as well as RAD51 activity, and to determine the role of this pathway in dysregulation of *RAD51* expression in malignancy.

## Materials and Methods

### 5′ and 3' Rapid Amplification of cDNA Ends (RACE) and reverse transcription.

5′ and 3' ends of *RAD51* and *TODRA* (*AK125393*) transcripts were determined using First Choice RLM-RACE kit (Ambion, Austin, TX, USA) on de-capped mRNA from HeLa cells, using the gene specific primers: (5'-AGCTGCCTCAGCCAGAATTTTA-3') and (5'-CCACACTGCTCTAACCGTGA-3'); (5′-ATGCGAGTAGGAGGCTCAGA-3′) and (5′-TACTGCCGAAACAAACCACA-3′) and (5′-AATAGTCCAGCTGCGATGGT-3′).

3'RACE used cDNA prepared from total RNA, using the primers: (5′-AGATAAACCTGGCCAACGTG-3′) and (5′-TGAACTCAGGAGGAGGTTGC-3′) or (5′-ATCGCCTTTCCAGTTTCTCA-3′) and (5′-CCCTACATTCCAATAACTCTACCTTC-3′) or (5′-TCTGCTCCTAATTCACCTCCTC-3′) and (5′-AAAACTAAGCCCAGCCGAAC-3′).

Strand-specific Reverse Transcription was performed using specific primers and SuperScript II (Invitrogen, Carlsbad, CA, USA) in the presence of RNase inhibitor (rRNasin, Promega, Madison, WI, USA).

### Cell culture

HeLa (ATCC, obtained from Ilana Keshet, the Hebrew University in Jerusalem), MCF7 (ATCC, obtained from Mary-Claire King, University of Washington, Seattle), and U2OS (ATCC) cells were cultured in DMEM, supplemented with 10% Fetal Bovine Serum (FBS), 2mM L-glutamine, 10U penicillin, 10μg streptomycin/ml (Biological Industries, Beit Ha'emek, Israel). Media for U2OS ER-E2F1 stable lines[42] also contained 0.5mg/ml G418 (Alexis Biochemicals, San Diego, CA, USA). Cell lines were maintained at 37°C with 5% CO_2_ and subcultured 2–3 times weekly.

ER-E2F1 induction of stably transfected U2OS cells, employed 300nM 4-hydroxytamoxifen (OHT) (Sigma-Aldrich, St Louis, MO, USA) following 48 hr. serum starvation (0.1% FBS containing medium).

### Plasmid construction

pRAD51-UTR, containing the *RAD51* promoter/5'UTR (-663 to +252, relative to the *RAD51* TSS) was cloned into pGL3-promoter (Promega). pRAD51, contains the *RAD51* core promoter (-114 to +15, relative to the *RAD51* TSS). pTODRA, contains the *TODRA* putative promoter (-320 to +40 relative to the TODRA TSS), in pGL3-basic (Promega). The bidirectional promoter (pBDP) was constructed by inserting the firefly luciferase gene into the Renilla luciferase reporter pGL4.70[hRluc] (Promega) in the opposite orientation. The *RAD51/TODRA* overlapping promoters were then amplified with the primers: (5'-CGTTCCATGGTACTCGGTCCGCAGCGCTC-3') and (5'-ATTCCATGGTCCCGTCTTGGGTTAGC-3'), and cloned between the two luciferase genes. The *TODRA* minigene was generated by PCR of gDNA using the primers (5'-CGCGTCGACGTAACGTATC-3') and (5'-ATTGCGGCCGCGAGACAAAGTTTCACTCTTTCG-3') and cloned downstream of an SV40 promoter in a pBluescript SK+ based vector.

Restriction and DNA modification enzymes were acquired from Fermentas MBI (Lithuania) and New England Biolabs (Beverly, MA, USA). QuickChange II site-directed mutagenesis kit (Stratagene, La Jolla, CA, USA) was used to introduce a 3bp mutation in the E2F site, confirmed by sequencing.

### Antibodies

The following primary antibodies were used in this study: polyclonal rabbit anti-E2F1 (C-20; Santa Cruz Biotechnology Cat# sc-193, RRID:AB_631394); monoclonal mouse anti-phospho-Histone H2A.X (Ser139), clone JBW301 (Millipore Cat# 05–636, RRID:AB_309864); and polyclonal rabbit anti-Rad51 (Ab-1; Merck Cat# PC130-100UL, RRID:AB_10684676). The following secondary antibodies were used in this study: DyLight 488 AffiniPure polyclonal Donkey anti Mouse IgG (H+L); and DyLight 594 monoclonal Donkey anti-rabbit IgG (minimal x-reactivity) (BioLegend Cat# 406405, RRID:AB_1575132).

### Transient transfections and luciferase activity measurement

MCF7 and HeLa cells were transfected in 50% confluent 24-well plates using jetPEI cationic polymer transfection reagent (Polyplus Transfection, Illkirch, France). Co-transfections with an E2F1 expression vector were preceded by 48 hr. serum starvation (with 0.1% FBS containing medium). Where indicated PTEN, TPIPα or TPIPβ pEGFP-C2 expression constructs were added (generous gift of Nicholas Leslie, University of Dundee).

Firefly and Renilla luciferase activities were quantitated sequentially using the Dual Luciferase Assay system (Promega) in a MiniLumat LB 9506 luminometer (EG&G Berthold, Germany). Where indicated, the pRL-TK, a Renilla luciferase plasmid driven by the HSV-thymidine kinase (TK) promoter (Promega), was used to normalize for transfection efficiencies by calculating the relative firefly/Renilla luciferase activities (RLA). Results of duplicates were averaged, and corrected for plasmid size differences to reflect equimolar measurements. Statistical analysis was performed using student's t-test.

### Quantitative Real Time RT-PCR

Total U2OS RNA was isolated using Tri-Reagent (Molecular Research Center Inc, Cincinnati, OH, USA) and reverse transcribed with ImpromII Reverse Transcriptase (Promega). The *TPIP* assay included a pre-amplification step using the TaqMan PreAmp Master Mix Kit (Applied Biosystems [ABI], Foster City, CA, USA). Real-time qPCR was performed using universal TaqMan or Power SYBR Green PCR master mix (ABI) in duplicates on the ABI PRISM 7900 Sequence Detector (ABI). Threshold cycle (Ct) values of the amplified genes were normalized to GAPDH levels, and relative expression levels were quantitated using the comparative method (User Bulletin #2, ABI PRISM 7700 Sequence Detection System, 1997) and calculated as 2^−ΔΔCt^.

All assays targeted RNA specific amplicons using either the TaqMan Gene Expression Assays Hs01685755_m1 for *TPIP*, or SYBR Green assays using the primers: *RAD51*: (5′-GCCCACAACCCATTTCAC-3′) and (5′-GGCAACAGCCTCCACAGTAT-3′), *TODRA*: (5′-TGATCCTGCGCGAGTTTACA-3′) and (5′-GGCCGACGCATTACCTCTT-3′) and *GAPDH*: (5′-CAGCCTCAAGATCATCAGCA-3′) and (5′-ACAGTCTTCTGGGTGGCAGT-3′).

### Chromatin immunoprecipitation (ChIP) assay

ChIP was performed using the EZ-ChIP kit (Millipore, Billerica, MA, USA). U2OS cells were cross-linked with 1% formaldehyde and sonicated. Immunoprecipitations used 1.5μg of polyclonal anti-E2F1 antibody (validated for ChIP in [59]) overnight at 4°C in parallel with no-antibody controls.

Quantitative analysis of ChIP assays was performed using real-time PCR in triplicates with Power SYBR Green, using the primers: (5′- GGAGGCGGGGATACGTTAC-3′) and (5′-CTCTCCTTAGGGCTCGGTC-3′). Calculations of Relative Promoter Occupancy were based on a previously described method[60], and calculated as: (1+Eff)^ΔCt^. Fold change in promoter occupancy was calculated between pairs of E2F1 induction/control: [(1+Eff) ^ΔCt^ OHT]/[(1+Eff) ^ΔCt^ control].

### mRNA expression Microarray

Total RNA extracted from two *TODRA* overexpressing and two control transfected Hela cell cultures (RNeasy Mini Kit, Qiagen, GmbH, Hilden, Germany) was assessed on a NanoDrop spectrophotometer (Thermo Fisher Scientific) and a 2100 Bioanalyzer (Agilent, Palo Alto, CA USA), amplified, fragmented and biotinylated as cDNA using the Affymetrix GeneChip Whole Transcript (WT) Sense Target Labeling Assay (Affymetrix, Santa Clara, CA) and hybridized to Affymetrix GeneChip Human Gene 1.0 ST expression arrays. The arrays were washed, stained, and scanned using the Affymetrix GeneChip Fluidics Station 400 and GeneChip Scanner 3000 7G.

### Microarray data analysis

Raw data was analyzed using the robust multiarray average (RMA) algorithm (Affymetrix Expression Console and Partek Genomics Suite 6.4)[61]. Raw intensity values were background corrected, log2 transformed, quantile normalized and a linear model was fit to the data to obtain an expression summary value for each probe set on each array. The data was analyzed using unsupervised hierarchical cluster analysis (Spotfire DecisionSite for Functional Genomics) and ANOVA (Partek) or student’s t-test. Fold change and p-value were used to identify differentially expressed genes. Complete microarray data are deposited at the Gene Expression Omnibus (GEO) database repository (NCBI) http://www.ncbi.nlm.nih.gov/geo/.

### HR Assay

DSB repair by HR was assessed in HR-inducible (HRind) U20S-DR-GFP cells stably transfected with an mCherry-*ISce*I-GR nuclease, as previously described[22]. In this study, HRind cells were grown in 6 well plates and transiently transfected with either the *TODRA* minigene, *TPIP* expression construct (tagged by mOrange2), or an appropriate empty vector. DSBs were induced by adding Dexamethasone to the growth medium 20–24 hours after transfection. Cells that were mCherry-positive (and mOrange2-positive in TPIP transfection experiments) or mCherry+GFP-positive (and mOrange2-positive in TPIP transfections) were counted 48 hours later by flow cytometry using the GACS ARIA III platform (Beckton Dickinson) and the GFP-positive fraction was calculated. Results of triplicates were averaged, and statistical analysis was performed using a student's t-test.

### Immunostaining and quantification of RAD51 foci

U2OS cells were grown on coverslips and transfected with either the *TODRA* minigene (pTODRA) or empty vector (pcDNA3). At 48 hours post-transfection, DNA damage was induced by adding phleomycin (10μg/ml) to the growth medium for 1 hour at 37°C. Cells were then either fixed immediately in 4% formaldehyde (time 0 post treatment, Fig 4C) or allowed to recover for 6 hours in fresh growth medium before fixation. For immunostaining, cells were permeabilized with 0.5% Triton X-100 and blocked in 2% BSA-PBS for 1 hour. After blocking, cells were probed with monoclonal anti-phospho-Histone H2A.X (Ser139)(1:300 dilution; validated for immunofluorescence in [62]) and polyclonal anti-RAD51 (1:300 dilution; validated for immunofluorescence in [63]) antibodies followed by secondary detection with donkey anti-mouse DyLight 488 (1:700 dilution) and donkey anti-rabbit DyLight 594 (1:700 dilution) fluorescent antibodies. For counterstaining, DNA was stained with 4′,6-diamidino-2-phenylindole (DAPI) and coverslips were then mounted onto slides with fluorescence mounting medium (Dover Medical). Fluorescent images were captured on an Olympus IX81 inverted microscope and processed off-line for foci counting using FiJi ImageJ software[64]. The number of RAD51-positive foci was normalized as the fraction of gamma-H2AX-positive foci per cell and averaged across all counted cells in each experimental condition. Results of triplicates were averaged, and statistical analysis was performed using student's t-test.

### Breast cancer tumors data analysis

We analyzed publicly available gene expression data from Muggerud *et al*.[28] including tumor samples from 31 cases of pure DCIS, 36 pure invasive cancers, 42 cases of mixed diagnosis (invasive cancer with an in situ component) and 6 normal breast tissue samples. In this study, global gene expression was assayed using the Agilent Whole Human Genome Oligo Microarrays 44k. Available data on samples included clinical information, e.g. age at onset, and pathology data, e.g. tumor grade, and ER, PR, ki67 and *HER2* amplification status. For each tumor sample, the relative expression of each gene (*RAD51*, *TODRA*, *TPIP* and *E2F1*), was normalized to the average expression of the same gene in the normal tissue samples. Correlation between expression levels of the different genes, and between expression levels and pathological and clinical information was performed using Pearson correlation for continuous variables and Spearman correlation and t-test for non-parametric comparisons (PASW Statistics 18).

## Supporting Information

S1 Fig E2F4 overexpression does not affect the *RAD51/TODRA* biderctional construct activity. pBDP activity was examined in MCF7 cells co-transfected with the pBDP construct and either an E2F4 or an empty expression vector. Results are depicted as the fold change between each E2F4 expression vector and the empty vector control, in the ratio of Firefly/Renilla luciferase activities, which represents the ratio of *RAD51/TODRA* promoter activities. Values are means ± SE of 3 independent transfections performed in duplicate.
(TIF)

S2 Fig *RAD51/TODRA* promoter occupancy of E2F1. E2F1 expression was induced in serum starved ER-E2F1 U2OS cells (stably transfected with a constitutively expressed ER-E2F1 fusion protein which upon ligand-dependent activation translocates from the cytoplasm to the nucleus) by treatment with OHT for 8 hours. *RAD51/TODRA* promoter occupancy was measured with a ChIP assay using E2F1 antibodies (Ab) in lysates of either OHT treated or untreated cells. Shown here is an unformatted representative gel of the promoter region PCR amplification products.
(TIF)
