# Supplementary figures and images for: TODRA, a lncRNA at the RAD51 Locus, Is Oppositely Regulated to RAD51, and Enhances RAD51-Dependent DSB (Double Strand Break) Repair
Source: PLoS One. 2015 Jul 31;10(7):e0134120. doi: 10.1371/journal.pone.0134120 (PMC4521930; doi:10.1371/journal.pone.0134120)

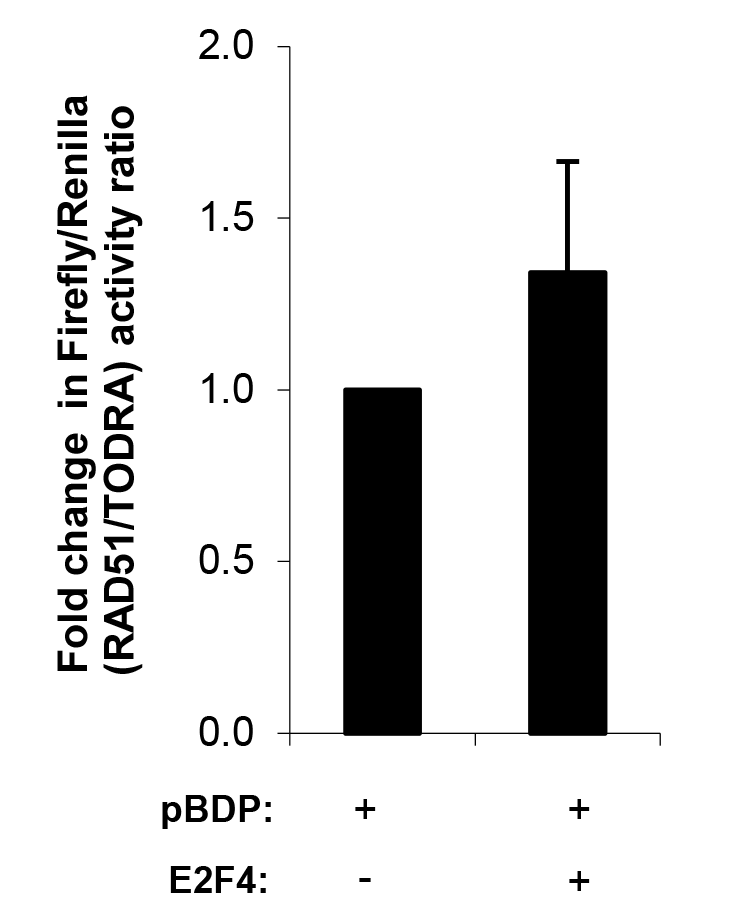

Supplement: S1 Fig — pBDP activity was examined in MCF7 cells co-transfected with the pBDP construct and either an E2F4 or an empty expression vector. Results are depicted as the fold change between each E2F4 expression vector and the empty vector control, in the ratio of Firefly/Renilla luciferase activities, which represents the ratio of RAD51/TODRA promoter activities. Values are means ± SE of 3 independent transfections performed in duplicate. (TIF) [file pone.0134120.s001.tif]

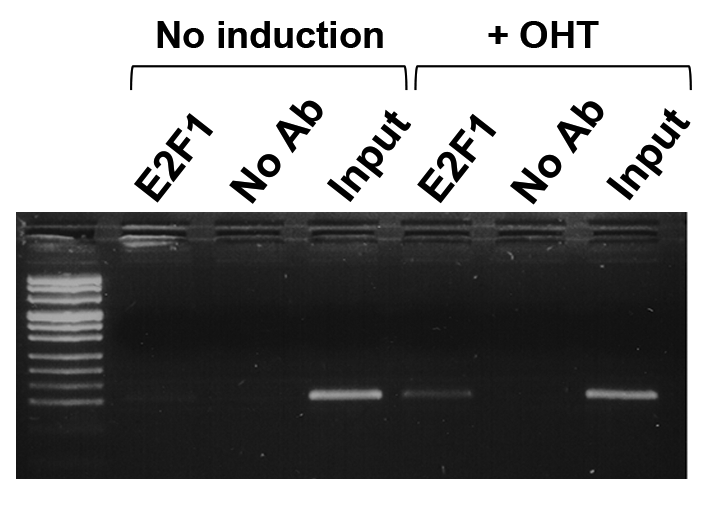

Supplement: S2 Fig — E2F1 expression was induced in serum starved ER-E2F1 U2OS cells (stably transfected with a constitutively expressed ER-E2F1 fusion protein which upon ligand-dependent activation translocates from the cytoplasm to the nucleus) by treatment with OHT for 8 hours. RAD51/TODRA promoter occupancy was measured with a ChIP assay using E2F1 antibodies (Ab) in lysates of either OHT treated or untreated cells. Shown here is an unformatted representative gel of the promoter region PCR amplification products. (TIF) [file pone.0134120.s002.tif]
